# Effect of 5-HT_7_ receptor blockade on liver regeneration after 60-70% partial hepatectomy

**DOI:** 10.1186/s12876-014-0201-2

**Published:** 2014-11-30

**Authors:** Konstantinos N Tzirogiannis, Kalliopi T Kourentzi, Sofia Zyga, Vassiliki Papalimneou, Maria Tsironi, Agni D Grypioti, Ioannis Protopsaltis, Dimitrios Panidis, Georgios I Panoutsopoulos

**Affiliations:** Department of Experimental Pharmacology, Medical School, Athens University, Athens, 11527 Greece; Department of Nursing, Faculty of Human Movement and Quality of Life Sciences, University of Peloponnese, Sparta, 23100 Greece; Department of Internal Medicine, Elpis General Hospital, Athens, 11522 Greece; Department of Internal Medicine, Tzanio General Hospital of Piraeus, Piraeus, 18537 Greece; Department of Nursing, Faculty of Human Movement and Quality of Life Sciences, University of Peloponnese, Orthias Artemidos and Plateon, Sparta, 23100 Greece

**Keywords:** Liver regeneration, Partial hepatectomy, 5-HT_7_ receptor, SB-269970, SB-258719, AS-19

## Abstract

**Background:**

Serotonin exhibits a vast repertoire of actions including cell proliferation and differentiation. The effect of serotonin, as an incomplete mitogen, on liver regeneration has recently been unveiled and is mediated through 5-HT_2_ receptor. The aim of the present study was to investigate the effect of 5-HT_7_ receptor blockade on liver regeneration after partial hepatectomy.

**Methods:**

Male Wistar rats were subjected to 60-70% partial hepatectomy. 5-HT_7_ receptor blockade was applied by intraperitoneal administration of SB-269970 hydrochloride two hours prior to and sixteen hours after partial hepatectomy and by intraperitoneal administration of SB-258719 sixteen hours after partial hepatectomy. Animals were sacrificed at different time points until 72 h after partial hepatectomy. Liver regeneration was evaluated by [^3^H]-thymidine incorporation into hepatic DNA, the mitotic index in hematoxylin-eosin (HE) sections and by immunochemical detection of Ki67 nuclear antigen. Reversion of 5-HT_7_ blockade was performed by intraperitoneal administration of AS-19. Serum and liver tissue lipids were also quantified.

**Results:**

Liver regeneration peaked at 24 h ([^3^H]-thymidine incorporation into hepatic DNA and mitotic index by immunochemical detection of Ki67) and at 32 h (mitotic index in HE sections) in the control group of rats. 5-HT_7_ receptor blockade had no effect on liver regeneration when applied 2 h prior to partial hepatectomy. Liver regeneration was greatly attenuated when blockade of 5-HT_7_ receptor was applied (by SB-258719 and SB-269970) at 16 h after partial hepatectomy and peaked at 32 h ([^3^H]-thymidine incorporation into hepatic DNA and mitotic index by immunochemical detection of Ki67) and 40 h (mitotic index in HE sections) after partial hepatectomy. AS-19 administration totally reversed the observed attenuation of liver regeneration.

**Conclusions:**

In conclusion, 5-HT_7_ receptor is a novel type of serotonin receptor implicated in hepatocyte proliferation.

## Background

Serotonin (5-HT) is an ancient chemical and neurotransmitter implicated in a vast variety of physiological and pathophysiological processes [1-3]. 5-HT mediates its actions through 14 distinct types of receptors encoded by a respective number of genes and its actions outnumber by far those of any other neurotransmitter. The majority of serotonin in the body (90%) is synthesized in the GI tract by enterochromafin cells and is known to control mood, behavior, memory, sleep and anxiety in the central nervous system (CNS). In the periphery, serotonin mediates vascular contraction and relaxation, GI tract smooth muscle cell tone (contraction and/or relaxation), platelet aggregation and is also acting as a growth factor for diverse cell types promoting survival, cell differentiation and proliferation as well as inhibition of apoptosis [1-3].

In the liver, serotonin is implicated in the regulation of blood flow at the level of portal vein and sinusoids through activation of 5-HT_2_ subtype of receptors [1], in biliary tree growth (5-HT_1α_ and 5-HT_1β_ receptors), in the development of liver cirrhosis through activation and proliferation of HSC cells (5-HT_2α_ and 5-HT_2β_) and hepatocyte proliferation (mainly 5-HT_2α/β_) [4]. Hepatocytes express SERT, 5-HT_2α_ and 5-HT_2β_ and possibly other types of serotonin receptors and HSC cells express 5-HT_1β_, 5-ΗΤ_1F_, 5-HT_2α_, 5-HT_2β_, 5-HT_7_ and SERT [1].

Reports regarding implication of serotonin in liver regeneration are dated back in the early 80s in non-English literature or even earlier [5,6]. A number of recent in vivo studies including studies from our laboratory have elucidated the role of serotonin in liver regeneration after partial hepatectomy [7-9] with platelets to be the major reservoir accounting for the increased hepatic concentrations of the monoamine during liver regeneration. From experiments with 5-HT_2_ receptor blockade with ketanserin or ritanserin in our laboratory, it has become evident that serotonin exerts its actions mainly at the G1/S transition point and this suggests implication of the monoamine in the control of this major restrictive checkpoint of the cell cycle [8]. In cultured rat hepatocytes, in in vitro experiments, serotonin induces dose-dependent increase in DNA synthesis only in the presence of insulin and epidermal growth factor (EGF) [7] and recently serotonin has been shown to promote hepatocellular cancer growth in human hepatocellular cancer cell lines [10].

5-HT_7_ receptor has been the last family of serotonin receptors to be discovered. It is a Gs coupled receptor with at least four different splice variants that differ in the length of the C termini and in the number of phosphorylation sites, and the above have significant biochemical consequences in the G protein coupling efficiency and the differential susceptibility to desensitization [11]. The distribution of the receptor has not been fully elucidated and its mRNA is most abundant in the thalamus, hippocampus and hypothalamus. In the central nervous system, 5-HT_7_ receptor mediates thermoregulation, learning and memory, regulation of circadian rhythms and mood, and endocrine functions. In the periphery the receptor is localized mainly on smooth muscle cells in blood vessels in a variety of organs where it mediates relaxation of blood vessels as well as in the gastrointestinal tract where it regulates motility [2,3,12].

In the present study, we investigated the effect of 5-HT_7_ receptor blockade on liver regeneration after partial hepatectomy.

## Methods

### Experimental animal model

Male Wistar rats, weighing 160–200 g, four to five months old (Hellenic Pasteur Institute, Athens, Greece) were used in this study. The animals were kept in a temperature-controlled room (22-25°C), under 12 h of light (08.00 h-20.00 h) and 12 h of darkness (20.00 h-08.00 h) and they had free access to a commercial pellet diet and tap water. The study protocol was approved by the Deontology Committee of the University of Peloponnese and animals were handled with humane care in accordance with the European Union Directive and adapted in the relevant Greek Presidential decree for the care and use of laboratory animals [13].

All surgical procedures were performed between 07.00-09.00 am with the animals under light ether anesthesia (diethyl ether per anesthesia; Codex, Carlo Erba, Milan, Italy). 5-HT_7_ receptor blockade was applied by intraperitoneal administration of SB-269970 hydrochloride (Sigma-Aldrich) and SB-258719 (Tokris Bioscience, Ellisville Missouri, USA). Reversion of 5-HT_7_ blockade was achieved by intraperitoneal administration of selective agonist AS-19 (Tokris Bioscience, Ellisville Missouri, USA).

The experimental rats were randomly assigned to the following groups:

- Group A: rats submitted to 60-70% partial hepatectomy and intraperitoneal administration of normal saline 2 h prior and 16 h after partial hepatectomy.
- Group B: rats submitted to 60-70% partial hepatectomy and intraperitoneal administration of SB-269970 hydrochloride at the dose of 2 mg/kg bodyweight 2 h prior to partial hepatectomy.
- Group C: rats submitted to 60-70% partial hepatectomy and intraperitoneal administration of SB-269970 hydrochloride at the dose of 2 mg/kg bodyweight 16 h after partial hepatectomy.
- Group D: rats submitted to 60-70% partial hepatectomy and intraperitoneal administration of SB-269970 hydrochloride at the dose of 2 mg/kg bodyweight 2 h prior and 16 h after partial hepatectomy.
- Group E: rats submitted to 60-70% partial hepatectomy and intraperitoneal administration of SB-258719 at the dose of 4 mg/kg bodyweight 16 h after partial hepatectomy.
- Group F: rats submitted to 60-70% partial hepatectomy, intraperitoneal administration of SB-269970 16 h after partial hepatectomy at the dose of 2 mg/kg bodyweight followed by intraperitoneal administration of AS-19 at the dose of 10 mg/kg bodyweight.
- Group G: rats submitted to 60-70% partial hepatectomy, intraperitoneal administration of SB-258719 16 h after partial hepatectomy at the dose of 4 mg/kg bodyweight followed by intraperitoneal administration of AS-19 at the dose of 10 mg/kg bodyweight.

**Figure 1:**
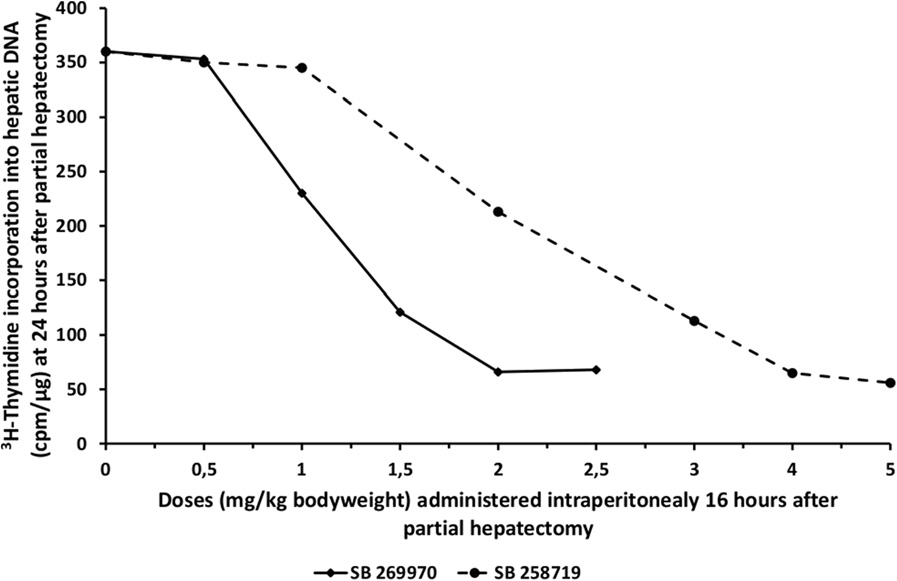
**Dose–response Curves of SB-269970 and SB-258719 administration.** Rate of liver regeneration at 24 h after 60-70% partial hepatectomy as evaluated by [^3^H]-thymidine incorporation into hepatic DNA in rats having administered different doses of SB-269970 (0.5, 1, 1.5, 2, and 2.5 mg/kg body weight) and SB-258719 (0.5, 1, 2, 4 and 5 mg/kg body weight) intraperitoneally at 16 h after partial hepatectomy. Results represent the findings from at least five rats. Values are expressed as means ± SE.

**Figure 2:**
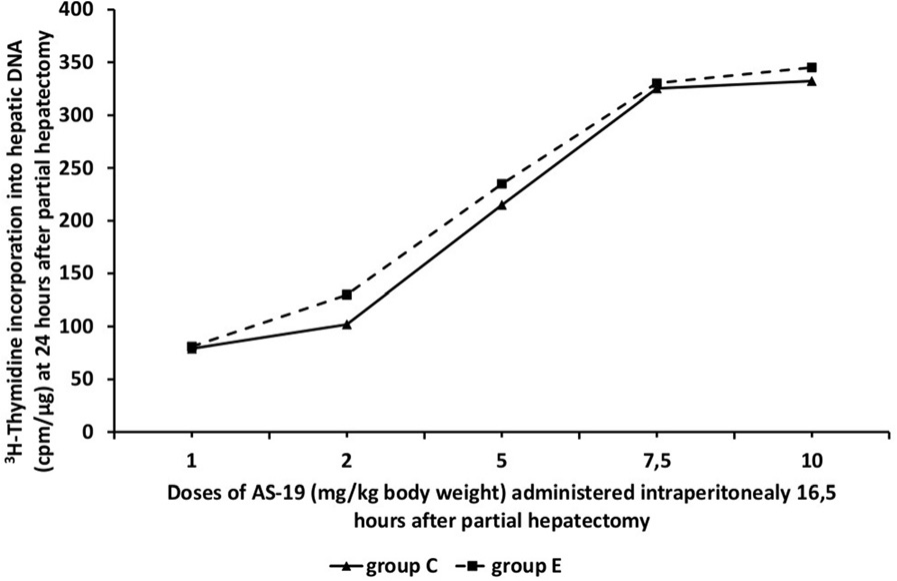
**Dose–response Curves of AS-19 administration.** Rate of liver regeneration at 24 h after 60-70% partial hepatectomy as evaluated by [^3^H]-thymidine incorporation into hepatic DNA in rats having administered SB-269970 hydrochloride (2 mg/kg bodyweight) 16 h after partial hepatectomy (group C) and SB-258719 (4 mg/kg bodyweight) 16 h after partial hepatectomy (group E) and different doses of AS-19 (1, 2, 5, 7.5 and 10 mg/kg body weight) intraperitoneally at 16.5 h after partial hepatectomy. Results represent the findings from at least five rats. Values are expressed as means ± SE.

Dosage of SB-269970 and SB-258719 was determined after dose–response experiments (Figure 1). Pilot experiments were also conducted with AS-19 (administration at the doses of 1, 2, 5, 7.5 and 10 mg/kg) (Figure 2).

Animals from groups A, B and D were killed at 8, 18, 20, 24, 32, 40, 48, 60 and 72 h after partial hepatectomy via cardiac puncture. Animals of groups C, E, F, and G were sacrificed at 18, 20, 24, 32, 40, 48, 60 and 72 h after partial hepatectomy.

One hour prior to sacrifice the animals of all groups were injected with [^3^H]-thymidine at the dose of 250 μCi/kg bodyweight intraperitoneally. A standard portion of the median liver lobe was used for histological evaluation and the rest was rapidly frozen in liquid nitrogen for further determinations. Liver weights were also tabulated for all groups of rats.

### Histological evaluation

A standard portion of the median liver lobe was fixed in 4% buffered formalin for 24 hours. Sections 5-μm thick were processed routinely, stained with hematoxylin-eosin (HE) and analysed for mitoses. Mitoses were counted in 10 randomly selected high-power fields (HPF) and expressed as the mean number of mitoses/HPF. The mitotic index was also evaluated by the immunochemical detection of Ki67 nuclear antigen (Dako, MIB 5 clone, 1:50, with microwave pre-treatment).

### Liver regeneration

The rate of liver regeneration was evaluated by the rate of [^3^H]-thymidine incorporation into hepatic DNA, the mitotic index in HE sections and by immunochemical detection of Ki67 nuclear antigen.

#### Rate of [^3^H]-thymidine Incorporation into Hepatic DNA

Animals of all groups were injected intraperitoneally with 250 μCi/kg bodyweight of [^3^H]-thymidine 1 h prior to sacrifice. DNA was extracted from the tissue according to the method of Munro and Fleck [14] as modified by Kyprianidis *et al.* [15]. The content of tissue DNA was estimated by the method of Richards [16]. The rate of [^3^H]-thymidine incorporation into hepatic DNA was calculated from the radioactivity measured in a liquid scintillation counter (Wallac LKB 1211 Rackbeta, Sweden) and results were expressed as counts/min/μg of DNA.

### Analysis of liver and serum lipid content

Frozen liver tissue (~100 mg) was homogenised in 1.6 ml phosphate-buffered saline and protein concentration was determined using the method of Lowry [17]. Lipids were extracted using chloroform: methanol (2:1) according to Folch et al. [18]. Phase separation was achieved with sulphuric acid 0.1% and the organic phase was solubilized in Triton X-100. Cholesterol, TG, FFA and phospholipid content were determined in liver tissue and plasma with the use of commercially available kits (Wako, Chemicals) and normalized to protein concentration of the homogenate. Free plasma glycerol levels were also determined in deproteinised serum samples as an indicator of lipolysis in adipose tissue [19].

### Statistical analysis

Data were expressed as means ± SE. All observations were obtained from at least five animals. The statistical analysis of the results was performed by unpaired Student’s *t*-test.

## Results

**Figure 3:**
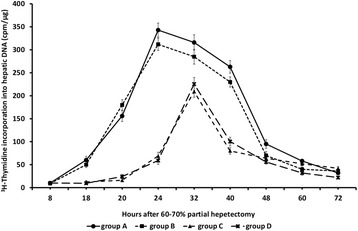
**Liver regeneration as evaluated by [** ^**3**^ **H]-thymidine incorporation into hepatic DNA in 60-70% partially hepatectomized rats and SB-269970.** Time course of liver regeneration as evaluated by [^3^H]-thymidine incorporation into hepatic DNA in 60-70% partially hepatectomized rats having received intraperitoneally saline (group A), SB-269970 hydrochloride (2 mg/kg bodyweight) 2 h prior to partial hepatectomy (group B), SB-269970 hydrochloride (2 mg/kg bodyweight) 16 h after partial hepatectomy (group C) or SB-269970 hydrochloride (2 mg/kg bodyweight) 2 h prior and 16 h after partial hepatectomy (group D). Results represent the findings from at least five rats: killed at 8, 18, 20, 24, 32, 40, 60 and 72 h (groups A, B and D) and at 18, 20, 24, 32, 40, 48, 60 and 72 h (group C). Values are expressed as means ± SE. DNA group A vs group C and D; P < 0.001: 18–40 h.

In rats subjected to 60-70% partial hepatectomy (group A), liver regeneration as evaluated by [^3^H]-thymidine incorporation into hepatic DNA, peaked at 24 and 32 h after partial hepatectomy and high rates were also observed at 40 h. The regenerative rates declined abruptly after 40 h and remained at low levels thereafter (Figure 3).

In rats subjected to 60-70% partial hepatectomy and intraperitoneal administration of SB-269970 2 h prior to partial hepatectomy (group B), [^3^H]-thymidine incorporation into hepatic DNA was maximal at 24 h and 32 h after partial hepatectomy with high rates also at 40 h (Figure 3). The temporal pattern and values of regenerative rate were almost identical in groups A and B of rats (Figure 3).

In group C of rats, intraperitoneal administration of SB-269970 16 h after partial hepatectomy greatly attenuated liver regeneration as evaluated by [^3^H]-thymidine incorporation into hepatic DNA at 24 h after partial hepatectomy (Figure 3). [^3^H]-thymidine incorporation into hepatic DNA was maximal at 32 h after partial hepatectomy in group C of rats and sharply declined thereafter (Figure 3). The maximal regenerative rate observed at 32 h in group C as well as the regenerative rates at all time points examined in this group were lower than the corresponding rates at the same time points for groups A and B (Figure 3).

In group D of rats [^3^H]-thymidine incorporation into hepatic DNA peaked at 32 h after partial hepatectomy showing the same temporal pattern as in group C (Figure 3). As in group C, liver regeneration was greatly attenuated at all time points examined.

**Figure 4:**
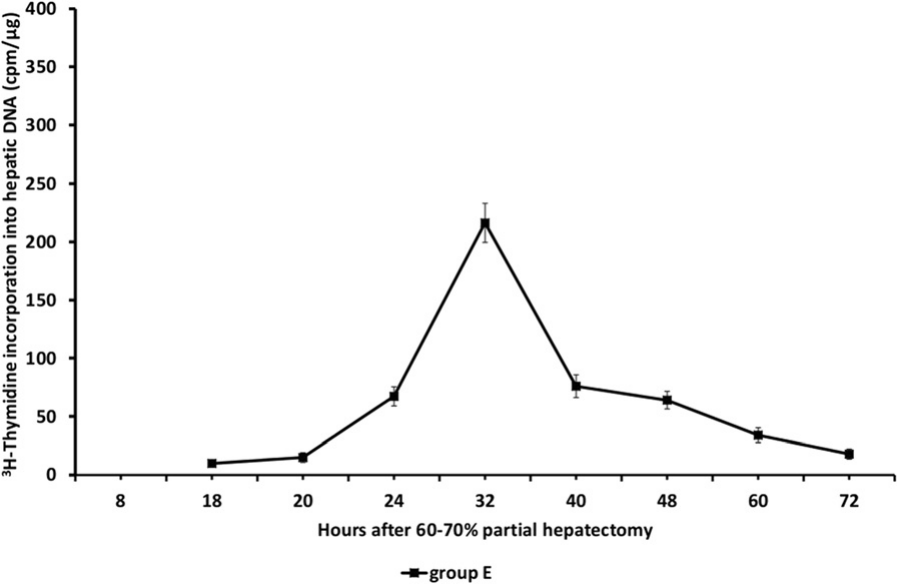
**Liver regeneration as evaluated by [** ^**3**^ **H]-thymidine incorporation into hepatic DNA in 60-70% partially hepatectomized rats and SB-258719.** Time course of liver regeneration as evaluated by [^3^H]-thymidine incorporation into DNA in 60-70% partially hepatectomized rats having received SB-258719 (4 mg/kg bodyweight) 16 h after partial hepatectomy. Results represent the findings from at least five rats killed at 18, 20, 24, 32, 40, 48, 60 and 72 h (group E). Values are expressed as means ± SE.

In group E of rats [^3^H]-thymidine incorporation into hepatic DNA peaked at 32 h after partial hepatectomy showing the same temporal pattern and similar values as in groups C and D (Figure 4). As in group C, liver regeneration was greatly attenuated at all time points examined.

**Figure 5:**
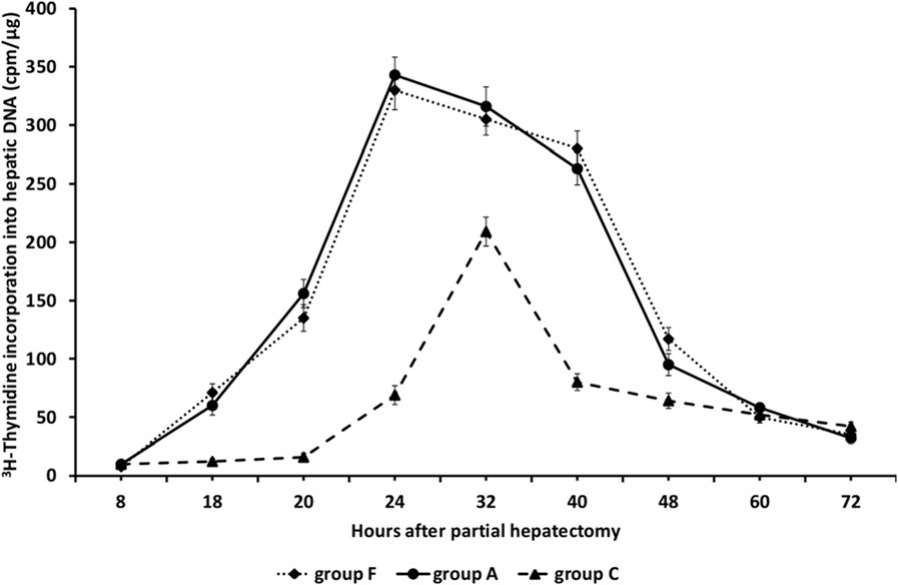
**Liver regeneration as evaluated by [** ^**3**^ **H]-thymidine incorporation into hepatic DNA in 60-70% partially hepatectomized rats and AS-19.** Time course of liver regeneration as evaluated by [^3^H]-thymidine incorporation into DNA in 60-70% partially hepatectomized rats having received intraperitoneally saline (group A) or SB-269970 hydrochloride (2 mg/kg bodyweight) 16 h after partial hepatectomy (group C) or SB-269970 hydrochloride (2 mg/kg bodyweight) followed by intraperitoneal administration of AS-19 (10 mg/kg bodyweight) 16.5 h after partial hepatectomy (group F). Results represent the findings from at least five rats killed at 8, 18, 20, 24, 32, 40, 48, 60 and 72 h (group A) and at 18, 20, 24, 32, 40, 48, 60 and 72 h (groups C and F). Values are expressed as means ± SE. DNA group C vs group F; P < 0.001: 18–48 h.

**Figure 6:**
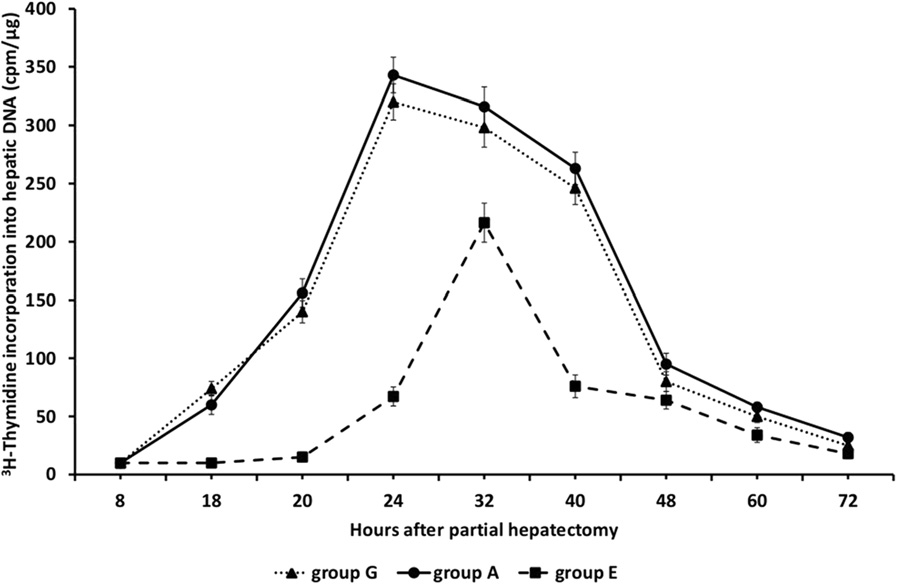
**Liver regeneration as evaluated by [** ^**3**^ **H]-thymidine incorporation into hepatic DNA in 60-70% partially hepatectomized rats and AS-19.** Time course of liver regeneration as evaluated by [^3^H]-thymidine incorporation into DNA in 60-70% partially hepatectomized rats having received intraperitoneally saline (group A) or SB-258719 (4 mg/kg bodyweight) 16 h after partial hepatectomy (group E) or SB-258719 (4 mg/kg bodyweight) followed by intraperitoneal administration of AS-19 (10 mg/kg bodyweight) 16.5 h after partial hepatectomy (group G). Results represent the findings from at least five rats killed at 8, 18, 20, 24, 32, 40, 48, 60 and 72 h (group A) and at 18, 20, 24, 32, 40, 48, 60 and 72 h (groups E and G). DNA group E vs group G; P < 0.001: 18–40 h.

In groups F and G, AS-19 administration reversed the observed attenuation of liver regeneration and [^3^H]-thymidine incorporation into hepatic DNA peaked at 24 and 32 h after partial hepatectomy while it was also at high levels at 40 h. The time pattern and values of [^3^H]-thymidine incorporation into hepatic DNA in groups F and G were almost identical with that in group A (Figures 5 and 6).

**Figure 7:**
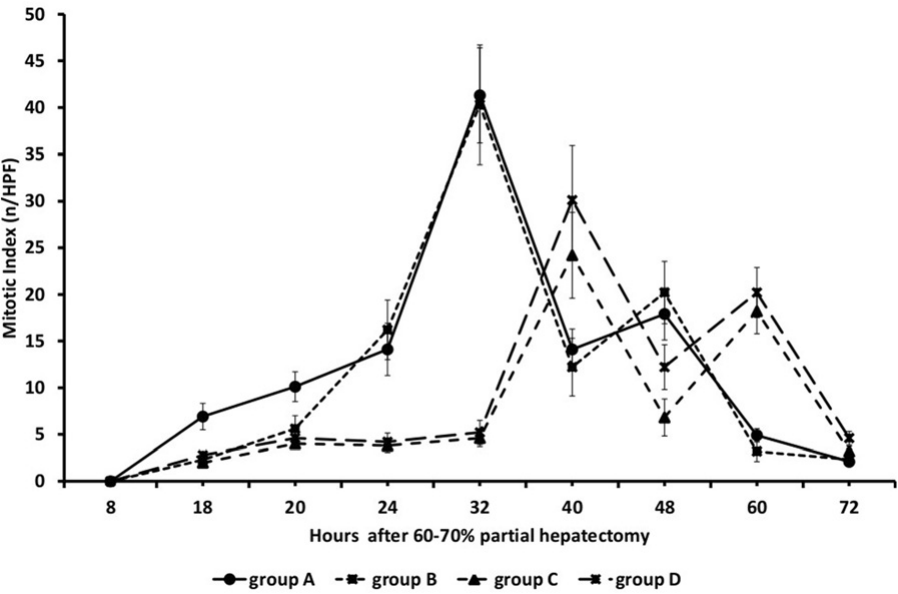
**Liver regeneration as evaluated by mitotic index (HE sections) in 60-70% partially hepatectomized rats and SB-269970.** Time course of liver regeneration as evaluated by mitotic index (HE sections) in 60-70% partially hepatectomized rats having received intraperitoneally saline (group A) or SB-269970 hydrochloride (2 mg/kg bodyweight) 2 h prior to partial hepatectomy (group B) or SB-269970 hydrochloride (2 mg/kg bodyweight) 16 h after partial hepatectomy (group C) or SB-269970 hydrochloride (2 mg/kg bodyweight) 2 h prior and 16 h after partial hepatectomy (group D). Mitotic index was expressed as mean number of mitoses/high-power field (HPF). Results represent the findings from at least five rats: killed at 8, 18, 20, 24, 32, 40, 60 h and 72 h (groups A, B and D) and at 18, 20, 24, 32, 40, 48, 60 and 72 h (group C). Values are expressed as means ± SE. Mitotic index group A vs group C and D; P < 0.001: 24–32 and 60 h; P < 0.01: 40 h. Mitotic index group A vs groups B, C and D; P < 0.01: 18 and 20 h. Mitotic index group A vs group C; P < 0.01: 48 h.

Mitotic index in HE sections was maximal at 32 h after partial hepatectomy in groups A and B with also relatively high levels at 24, 40 and 48 h and sharply declined thereafter (Figure 7). In groups C and D of rats, mitotic index was minimal until 32 h and two major peaks were observed at 40 and 60 h that were both lower than the corresponding peaks in groups A and B at 32 h (Figure 7).

**Figure 8:**
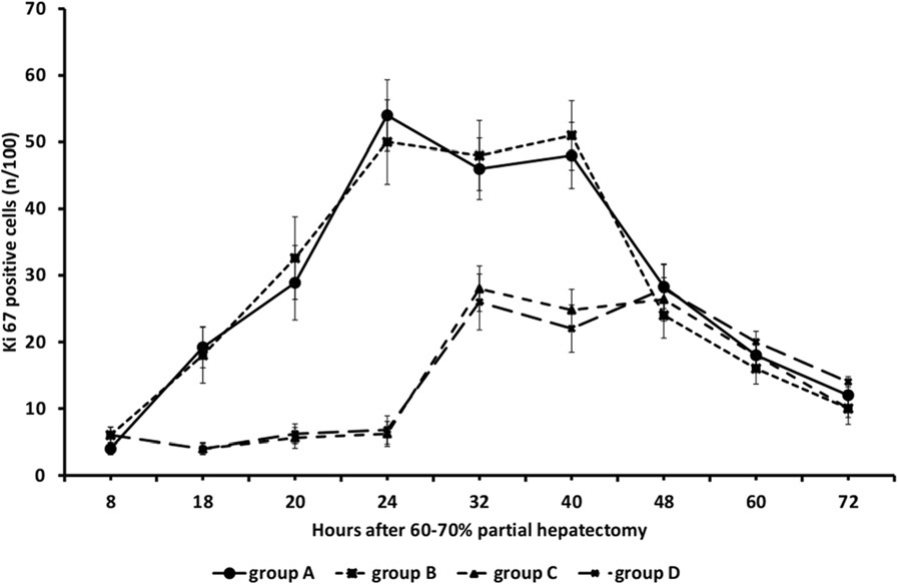
**Ki67 positive cells in 60-70% partially hepatectomized rats and SB-269970.** Time course of Ki67 positive cells in 60-70% partially hepatectomized rats having received intraperitoneally saline (group A) or SB-269970 hydrochloride (2 mg/kg bodyweight) 2 h prior to partial hepatectomy (group B) or SB-269970 hydrochloride (2 mg/kg bodyweight) 16 h after partial hepatectomy (group C) or SB-269970 hydrochloride (2 mg/kg bodyweight) 2 h prior and 16 h after partial hepatectomy (group D). Results represent the findings from at least five rats killed at 8, 18, 20, 24, 32, 40, 48, 60 h and 72 h (groups A, B and D) at 18, 20, 24, 32, 40, 48, 60 and 72 h (group C). Values are expressed as means ± SE. Ki67 group A vs group C and D; P < 0.001: 18–40 h.

**Figure 9:**
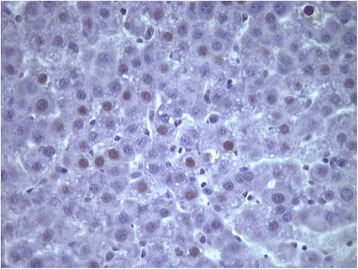
**Ki67 positive cells at 24 h (×400) in 60–70% partially hepatectomized rats having received saline (group A).**

**Figure 10:**
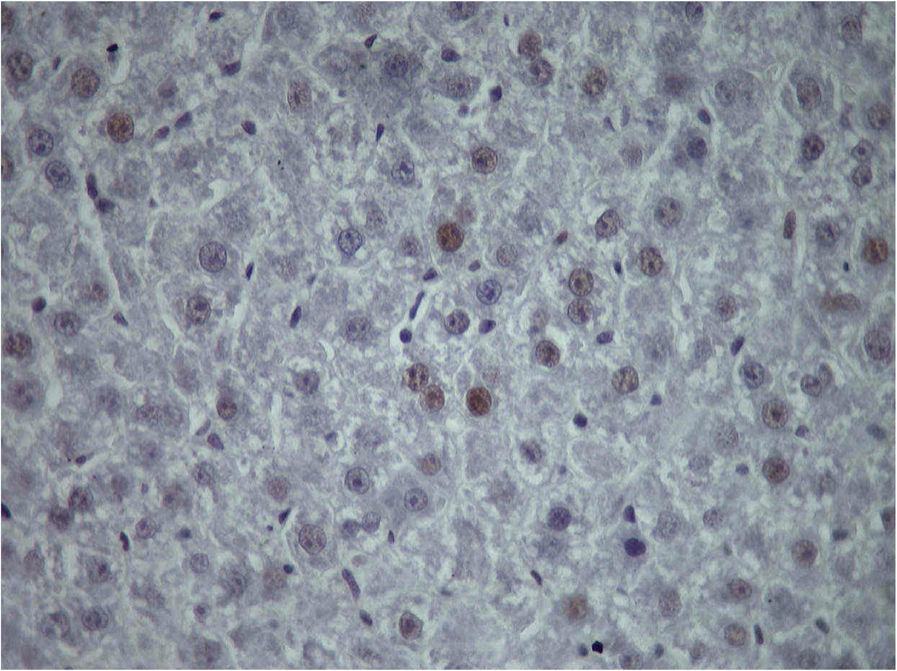
**Ki67 positive cells at 24 h (×400) in 60–70% partially hepatectomized rats having received SB-269970 hydrochloride (2 mg/kg bodyweight) 16 h after partial hepatectomy (group C).**

Mitotic index as evaluated by the immunochemical detection of Ki67 gradually increased between 8 and 24 h when it peaked in groups A and B of rats and remained at high levels until 40 h with abrupt decline thereafter (Figures 8 and 9). The index remained at low levels between 8 and 24 h after partial hepatectomy in groups C and D of rats with sharp increase at 32 h (Figures 8 and 10). The percentage of Ki67 nuclei remained at relatively high levels until 48 h after partial hepatectomy with gradual decrease afterwards in these groups of rats (Figure 8). The regenerative rate as evaluated by Ki67 positive cells in groups C and D at 32 and 40 h was lower than that in groups A and B.

**Figure 11:**
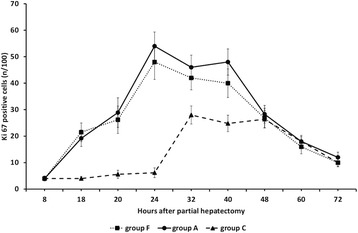
**Ki67 positive cells in 60-70% partially hepatectomized rats and AS-19.** Time course of Ki67 positive cells in 60-70% partially hepatectomized rats having received intraperitoneally saline (group A) or SB-269970 hydrochloride (2 mg/kg bodyweight) 16 h after partial hepatectomy (group C) or SB-269970 hydrochloride (2 mg/kg bodyweight) followed by intraperitoneal administration of AS-19 (10 mg/kg bodyweight) 16.5 h after partial hepatectomy (group F). Results represent the findings at least five rats killed at 8, 18, 20, 24, 32, 40, 48, 60 and 72 h (group A) and at 18, 20, 24, 32, 40, 48, 60 and 72 h (groups C and F). Values are expressed as means ± SE. Ki67 group C vs group F; P < 0.001: 18–40 h.

In group F intraperitoneal administration of AS-19 at the dose of 10 mg/kg of body weight totally reversed the observed attenuation of liver regeneration as evaluated by the percentage of Ki67 positive cells and regenerative rates were almost identical with these in group A (Figure 11). The observed effect of AS-19, as evaluated in initial pilot experiments was dose-dependent (Figure 2). In group G of rats AS-19 administration also totally reversed the observed inhibition of liver regeneration and the time pattern and values of Ki67 positive cells were also almost identical with these in groups A and F (data not shown).

Relative liver weight (liver weight in g/100 g bodyweight) sharply decreased, as expected after partial hepatectomy, with gradual increase thereafter in group A of rats. In groups C, D and E, relative liver weight remained at low levels without significant increases until 24 h after partial hepatectomy. In these groups a small increase was observed at 32 h with further increase at 40 and 48 h after partial hepatectomy. In groups F and G of rats, the relative liver weights showed the same gradual increases as in group A (Table 1).

**Table 1:** **Relative liver weights (g/100 g bodyweight) after 60-70% partial hepatectomy in groups A, C, E, F and G**

	**Relative liver weights (g/100 g body weight)**				
**Hours after partial hepatectomy**	**Group A**	**Group C**	**Group E**	**Group F**	**Group G**
8	1,6 ± 0,1	1,5 ± 0,1	1,6 ± 0,1	1,7 ± 0,2	1,6 ± 0,1
18	1,9 ± 0,2	1,7 ± 0,1	1,6 ± 0,1	2,0 ± 0,2	2,0 ± 0,1
24	2,3 ± 0,2	1,7 ± 0,1	1,8 ± 0,1	2,2 ± 0,2	2,4 ± 0,2
32	2,6 ± 0,3	2,3 ± 0,2	2,2 ± 0,2	2,5 ± 0,3	2,7 ± 0,2
40	3,1 ± 0,3	2,6 ± 0,2	2,6 ± 0,1	3,1 ± 0,2	3,0 ± 0,3
48	3,5 ± 0,2	2,7 ± 0,2	2,8 ± 0,2	3,4 ± 0,3	3,4 ± 0,3
60	3,7 ± 0,2	2,7 ± 0,2	2,7 ± 0,1	3,8 ± 0,2	3,7 ± 0,3
72	4,2 ± 0,2	2,6 ± 0,1	2,8 ± 0,1	4,1 ± 0,3	4,3 ± 0,2

The mean relative liver weight for normal rats (n = 5) of the same age and weight range was 4.5 ± 0.3.

Regarding lipid changes after partial hepatectomy, increase in liver triglyceride levels was observed at 18 h after partial hepatectomy with further increase at 24 h in group A of rats. Liver triglyceride content peaked at 40 h after partial hepatectomy and decreased thereafter but was still at high levels at 72 h after partial hepatectomy. Serum triglyceride concentration decreased at 18 and 24 h after partial hepatectomy and increased afterwards and these increases were still present at 72 h after partial hepatectomy. Serum FFA and free glycerol levels both increased at 18 h after partial hepatectomy and remained at high levels thereafter. The temporal patterns of liver and plasma lipid changes were similar in all groups of rats (Tables 2 and 3).

**Table 2:** **Liver and serum triacylglycerol levels and serum glycerol and FFA levels in groups A, C and D**

**Time after PH (hours)**	**Group A**				**Group C**				**Group D**			
	**Liver triacylglycerol (μg/mg of protein)**	**Serum triacylglycerol (mg/dl)**	**Serum glycerol (μmol/l)**	**Serum FFA (μmol/ml or mmol/l)**	**Liver triacylglycerol (μg/mg of protein**	**Serum triacylglycerol (mg/dl)**	**Serum glycerol (μmol/l)**	**Serum FFA (μmol/ml or mmol/l)**	**Liver triacylglycerol (μg/mg of protein)**	**Serum triacylglycerol (mg/dl)**	**Serum glycerol (μmol/l)**	**Serum FFA (μmol/ml or mmol/l)**
0	15.8 ± 0.8	6.2 ± 0.6	61.2 ± 5.2	0.32 ± 0.05	15.8 ± 0.8	6.2 ± 0.6	61.2 ± 5.2	0.32 ± 0.05	15.8 ± 0.8	6.2 ± 0.6	61.2 ± 5.2	0.32 ± 0.05
8	16.8 ± 0.9	5.8 ± 0.6	75.4 ± 6.5	0.44 ± 0.05	N.D.	N.D.	N.D.	N.D.	17.4 ± 1.3	6.0 ± 0.9	72.8 ± 6.1	0.50 ± 0.06
18	28.1 ± 2.3	3.8 ± 0.4	188.6 ± 8.8	0.86 ± 0.07	27.5 ± 3.1	4.2 ± 0.4	174.2 ± 7.5	0.82 ± 0.08	29.4 ± 2.4	3.5 ± 0.6	179.4 ± 7.8	0.89 ± 0.08
20	30.6 ± 3.4	3.6 ± 0.5	192.2 ± 9.5	0.84 ± 0.08	29.7 ± 2.5	3.9 ± 0.4	190.3 ± 8.6	0.86 ± 0.09	31.2 ± 2.6	3.3 ± 0.4	195.1 ± 8.2	0.81 ± 0.06
24	34.8 ± 3.8	3.4 ± 0.4	187.8 ± 9.1	0.82 ± 0.07	35.3 ± 3.4	3.2 ± 0.5	195.2 ± 8.9	0.89 ± 0.09	36.2 ± 2.9	3.0 ± 0.4	189.3 ± 8.8	0.79 ± 0.07
32	37.2 ± 2.3	5.6 ± 0.6	204.2 ± 10.4	0.90 ± 0.06	38.1 ± 3.8	4.8 ± 0.4	197.5 ± 9.3	0.94 ± 0.08	37.8 ± 2.7	5.4 ± 0.7	201.8 ± 9.4	0.88 ± 0.09
40	40.1 ± 3.4	6.4 ± 0.7	196.3 ± 10.1	0.86 ± 0.09	41.3 ± 4.2	5.9 ± 0.6	203.4 ± 8.7	0.88 ± 0.07	39.2 ± 3.1	6.7 ± 0.8	204.9 ± 8.5	0.91 ± 0.09
48	33.8 ± 2.6	7.4 ± 0.7	205.1 ± 9.5	0.84 ± 0.08	34.5 ± 3.5	7.1 ± 0.7	197.8 ± 9.5	0.84 ± 0.08	31.6 ± 2.5	7.8 ± 0.9	209.5 ± 9.7	0.80 ± 0.08
60	26.6 ± 2.2	7.2 ± 0.8	179.9 ± 8.6	0.83 ± 0.07	27.6 ± 3.1	7.4 ± 0.6	189.7 ± 7.8	0.81 ± 0.08	27.3 ± 1.9	7.7 ± 0.6	192.5 ± 8.3	0.78 ± 0.08
72	24.6 ± 1.6	7.0 ± 0.6	204.1 ± 8.9	0.85 ± 0.08	26.5 ± 2.6	7.5 ± 0.7	196.8 ± 7.5	0.80 ± 0.07	23.2 ± 1.7	7.2 ± 0.8	189.6 ± 7.8	0.74 ± 0.06

Values are expressed as mean ± standard error.

FFA = Free fatty acid.

N.D. = Not Determined.

**Table 3:** **Liver and serum triacylglycerol levels and serum glycerol and FFA levels in groups E, F and G**

**Time after PH (hours)**	**Group E**				**Group F**				**Group G**			
	**Liver triacylglycerol (μg/mg of protein)**	**Serum triacylglycerol (mg/dl)**	**Serum glycerol (μmol/l)**	**Serum FFA (μmol/ml or mmol/l)**	**Liver triacylglycerol (μg/mg of protein**	**Serum triacylglycerol (mg/dl)**	**Serum glycerol (μmol/l)**	**Serum FFA (μmol/ml or mmol/l)**	**Liver triacylglycerol (μg/mg of protein)**	**Serum triacylglycerol (mg/dl)**	**Serum glycerol (μmol/l)**	**Serum FFA (μmol/ml or mmol/l)**
0	15.8 ± 0.8	6.2 ± 0.6	61.2 ± 5.2	0.32 ± 0.05	15.8 ± 0.8	6.2 ± 0.6	61.2 ± 5.2	0.32 ± 0.05	15.8 ± 0.8	6.2 ± 0.6	61.2 ± 5.2	0.32 ± 0.05
18	30.3 ± 2.9	4.0 ± 0.6	180.7 ± 8.3	0.85 ± 0.09	28.9 ± 3.3	3.8 ± 0.5	175.9 ± 8.5	0.72 ± 0.08	29.9 ± 2.8	4.2 ± 0.8	184.8 ± 8.8	0.81 ± 0.08
20	32.7 ± 3.8	3.7 ± 0.5	191.3 ± 8.9	0.83 ± 0.06	30.8 ± 3.5	3.9 ± 0.5	190.7 ± 9.0	0.79 ± 0.09	34.3 ± 3.1	3.6 ± 0.7	196.7 ± 8.5	0.77 ± 0.08
24	35.7 ± 3.9	3.5 ± 0.5	194.5 ± 9.7	0.80 ± 0.07	34.4 ± 3.8	3.4 ± 0.7	193.8 ± 8.5	0.87 ± 0.13	37.2 ± 3.5	3.1 ± 0.6	199.5 ± 9.2	0.79 ± 0.07
32	39.5 ± 3.3	5.0 ± 0.9	201.6 ± 10.3	0.93 ± 0.11	42.3 ± 4.0	5.3 ± 0.9	198.5 ± 9.9	0.96 ± 0.15	38.8 ± 3.7	4.5 ± 0.9	207.8 ± 10.4	0.90 ± 0.09
40	42.6 ± 4.1	6.7 ± 0.6	199.2 ± 10.8	0.86 ± 0.09	44.6 ± 4.6	6.3 ± 1.1	205.6 ± 10.7	0.91 ± 0.09	43.4 ± 3.6	6.1 ± 0.9	210.9 ± 9.5	0.95 ± 0.09
48	35.9 ± 2.9	7.7 ± 0.8	206.7 ± 11.3	0.83 ± 0.08	37.5 ± 3.9	7.6 ± 0.9	203.7 ± 10.2	0.87 ± 0.08	32.9 ± 3.5	7.2 ± 1.2	200.3 ± 10.7	0.81 ± 0.08
60	27.4 ± 2.6	7.1 ± 0.9	182.9 ± 9.6	0.81 ± 0.08	29.8 ± 3.3	7.4 ± 0.9	185.8 ± 8.8	0.82 ± 0.07	26.3 ± 2.4	7.3 ± 0.9	172.7 ± 8.9	0.73 ± 0.07
72	23.6 ± 2.2	6.7 ± 0.6	200.4 ± 9.9	0.85 ± 0.09	25.6 ± 2.9	6.5 ± 0.8	203.6 ± 9.5	0.76 ± 0.09	21.8 ± 1.9	7.0 ± 1.0	192.6 ± 7.5	0.81 ± 0.09

Values are expressed as mean ± standard error.

FFA = Free fatty acid.

## Discussion

The ability of the liver to regenerate after surgical resection or any short of hepatic injury has been known from long and has drawn immense scientific interest. 60-70% partial hepatectomy is the most commonly applied stimulus for the study of liver regeneration mainly due to the fact that the mitotic stimulus is accurately applied in time and not associated with necrotic or inflammatory processes [20]. A great number of substances influence the regenerative process and traditionally they are classified as complete and incomplete (auxiliary) mitogens [20].

The autonomic nervous system, both sympathetic and parasympathetic, is implicated in liver regeneration although the exact mechanisms of its effects still remain obscure [21-23]. Among neurotransmitters norepinephrine, mainly through α_1_-adrenergic receptor [23-25] (actions through β-adrenergic receptors have also been reported) [26], and serotonin, mainly through 5-HT_2_ receptor, are considered auxiliary mitogens [7-9].

Serotonin is an important neurotransmitter of the autonomic nervous system and in the liver serotonergic nerve fibres are localized in the tunica media of branches of the hepatic artery, portal vein, bile ducts and the connective tissue of the interlobular septae in humans and rats [27,28]. 5-HT receptors are expressed in various liver cell types, apart from hepatocytes, as hepatic stelate cells and sinusoidal endothelial cells [4,29,30].

From experiments on differential 5-HT receptor subtype expression and blockade experiments with various receptor antagonists of other research groups it has become evident that 5-HT_2α_ and 5-HT_2β_ receptors mediate liver regeneration [31] and molecular pathways have been elucidated in the case of 5-HT_2β_ receptor [32-34].

In our study, 5-HT_7_ receptor blockade greatly attenuated liver regeneration when applied close to the G_1/_S transition point of the cell cycle and this is the first study to reveal implication of the 5-HT_7_ receptor in liver regeneration and more specifically in this major restrictive cell cycle check point. In the central nervous system, blockade of 5-HT_7_ receptor has been reported to increase hippocampal cell proliferation [35] and the receptor is also implicated at least in the initial stages of T-cell activation and possibly in T-cell proliferation [36]. Additionally, 5-ΗΤ_7_ receptor has been recently found to be expressed in hepatocytes although the full repertoire of its actions in the liver still remains obscure [37].

SB-269970 used in our study is considered a highly selective ligand for 5-HT_7_ receptors (pKi= 8.9 ± 0.1) with at least 100-fold greater affinity in relation to other types of 5-HT receptor subtypes but some researchers have also reported that it is also a potent α_2_-adrenergic receptor blocker [38-41]. Although only α_1_-adrenoreceptors have been reported to participate in liver regeneration, the observed inhibitory effect by SB-269970 could also be attributed to α_2_-receptor blockade especially since α_2_-adrenoreceptors are expressed in hepatocytes [42,43]. Activation of α_2_-adrenergic receptors has been reported to induce cell proliferation in different cell types [44-46], whereas competitive inhibition of these receptors attenuates cell proliferation and/or induces apoptosis [44,45,47]. However, there are reports that connect α_2_-receptor stimulation with inhibition of cell growth [48]. In order to elucidate the above, another series of experiments has been conducted in our laboratory with intraperitoneal administration of SB-258719 (pKi= 7.5) at the dose of 4 mg/kg bodyweight at 16 h after partial hepatectomy [38,49,50]. SB-258719 is a known weak inverse agonist of 5-HT_7_ receptor without any known actions on other type of serotonin receptors and its administration had the same effect on liver regeneration as SB-269970 administration and the above clearly suggests that the observed inhibitory effect must be attributed to 5-HT_7_ receptor blockade.

In order to verify that the observed effect on liver regeneration is due to blockade of 5-HT_7_ receptor we conducted another series of experiments with the selective 5-HT_7_ receptor agonist AS-19 [51-53]. AS-19 is considered a selective 5 HT_7_ agonist (K*i* = 0.6 nM, IC_50_ = 0.83nM) [54]. AS-19 administration reversed the observed attenuation of liver regeneration caused by administration of SB-269970 and SB-258719 and this verifies the implication of 5-HT_7_ in liver regeneration.

It is known from long that liver regeneration is accompanied by transient hepatic steatosis and intracellular accumulation of triglycerides in hepatocytes through increased lipolysis in the adipose tissue and increased hepatic lipogenesis [55,56]. Serotonin induces lipolysis in adipocytes and promotes gluconeogenesis in hepatocytes through 5-HT_2b_ receptor during fasting adaptation [57]. Additionally serotonin is also implicated in the regulation of lipid metabolism through 5-HT_2c_ receptors by altering sympathetic outflow at the brain level [58]. In our experiments no significant differences have been observed in serum and liver lipids during liver regeneration after 5-HT_7_ receptor blockade and consequently 5-HT_7_ receptor does not seem to be implicated in the adaptive changes of lipid metabolism during liver regeneration.

5-HT_7_ receptors have been reported to activate MAPK [59,60] and this activation has also been reported to be RAS-dependent [61]. The above seems to represent a more general pattern of MAPK activation from Gs- coupled receptors with RAS independent pathways to have also been described [62,63]. Both 5-HT_2α_ and 5-HT_2β_ receptors have also been reported to activate MAPK through similar pathways [33,64] and this hints at a possible role of 5-HT_7_ receptor in mitogenesis and cell-cycle progression although further research is needed at this point.

## Conclusions

The results of this study indicate that 5-HT_7_ receptor is implicated in liver regeneration after partial hepatectomy. Serotonin through 5-HT_7_ receptor seems to exert its auxiliary proliferative effect close to G1/S transition point and during the S phase. Therefore, the results identify a novel type of 5-HT receptor that mediates the proliferative effect of the monoamine in the liver.

## Abbreviations

5-HT: Serotonin
HE: Hematoxylin-eosin
CNS: Central nervous system
GI: Gastro-intestinal
EGF: Epidermal growth factor
HPF: High-power fields
MAPK: Mitogen-activated protein kinase

## References

[1] Ruddell RG, Mann DA, Ramm GA (2008). The function of serotonin within the
liver. J Hepatol.

[2] Beattie DT, Smith JA (2008). Serotonin Pharmacology in the gastrointestinal tract:
a review. Naunyn Schmiedebergs Arch Pharmacol.

[3] Lesurtel M, Soll C, Graf R, Clavien PA (2008). Role of serotonin in the hepato-gastrointestnal tact:
an old molecule for new perspectives. Cell Mol Life Sci.

[4] Ruddel RG, Oakley F, Hussain Z, Yeung I, Bryan-Lluka LJ, Ramm GA, Mann DA (2006). A Role for Serotonin (5-HT) in hepatic Stellate Cell
Function and Liver Fibrosis. Am J Pathol.

[5] Kulinskii VI, Saratikov AS, Vstavskaia IA, Udovitsina TI (1983). Receptors mediating serotonin-stimulating liver
regeneration in mice. Biull Eksp Biol Med.

[6] Kulinskii VI, Udovitsina TI, Vstavskaia IA, Rykov SA (1983). Comparison of the changes in mitotic activity and in
serotonin concentration in regenerating liver. Vopr Med Khim.

[7] Balasubramanian S, Paulose CS (1998). Induction of DNA synthesis in primary cultures of rat
hepatocytes by serotonin: possible involvement of serotonin S2
receptor. Hepatology.

[8] Papadimas GK, Tzirogiannis KN, Panoutsopoulos GI, Demonakou MD, Skaltsas SD, Hereti RI, Papadopoulou-Daifoti Z, Mykoniatis MG (2006). Effect of serotonin receptor 2 blockade on liver
regeneration after partial hepatectomy in the rat liver. Liver Int.

[9] Lesurtel M, Graf R, Aleil B, Walther DJ, Tian Y, Jochum W, Gachet C, Bader M, Clavien PA (2006). Platelet-derived serotonin mediates liver
regeneration. Science.

[10] Soll C, Jang JH, Riener MO, Moritz W, Wild PJ, Graf R, Clavien PA (2010). Serotonin promotes tumor growth in Human
Hepatocellular Cancer. Hepatology.

[11] Vanhoenacker P, Haegeman G, Leysen JE (2000). 5-HT7 receptors: current knowledge and future
prospects. Trends Pharmacol Sci.

[12] Hedlund PB, Sutcliffe JG (2004). Functional, molecular and pharmacological advances in
5-HT 7 receptor research. Trends Pharmacol Sci.

[13] GREEK PRESIDENTIAL DECREE No
160/1991 (1991). Protection of Animals Used for Experimental and Other Scientific
Purposes in Accordance With EU Directive 86/609/EEC of the Council.

[14] Munro HN, Fleck A (1966). Recent developments in the measurement of nucleic
acids in biological materials. Analyst.

[15] Kyprianidis KG, Mykoniatis MG, Papadimitriou DG, Valsamidou A (1996). Effect of subtotal pancreatectomy on the rate of liver
regeneration: the role of hepatic stimulator substance. J Surg Res.

[16] Richards G (1974). Modifications of the diphenylamine reaction giving
increased sensitivity and simplicity in the estimation of DNA. Anal Biochem.

[17] Lowry O, Rosenbrough N, Farr A, Randall R (1951). Protein measurement with the folin phenol
reagent. J Biol Chem.

[18] Folch J, Lees M, Sloane Stanley GH (1982). A simple method for the isolation and purification of
total lipids from animal tissues. J Biol Chem.

[19] Tijburg LB, Maquedano A, Bijleveld C, Guzman M, Geelen MJ (1988). Effects of ethanol feeding on hepatic lipid
synthesis. Arch Biochem Biophys.

[20] Michalopoulos GK (2010). Liver regeneration after partial Hepatectomy: critical
analysis of mechanistic dilemmas. Am J Pathol.

[21] Tanaka K, Ohkawa S, Nishino T, Niijima A, Inoue S (1987). Role of the hepatic branch of the vagus nerve in lever
regeneration in rats. Am J Physiol.

[22] Oben JA, Diehl AM (2004). Sympathetic nervous system regulation of liver
repair. Anat Rec A: Discov Mol Cell Evol Biol.

[23] Cruise JL, Knechtle SJ, Bollinger RR, Kuhn C, Michalopoulos G (1987). Alpha 1-adrenergic effects and liver
regeneration. Hepatology.

[24] Cruise JL, Houck KA, Michalopoulos G (1988). Early events in the regulation of Hepatocyte DNA
synthesis: the role of alpha- adrenergic stimulation. Scand J Gastroenterol.

[25] Cruise JL, Houck KA, Michalopoulos GK (1985). Induction of DNA synthesis in cultured rat hepatocytes
through stimulation of alpha 1 adrenoreceptor by norepinephrine. Science.

[26] Refsnes M, Thoresen GH, Sandnes D, Dajani OF, Dajani L, Christoffersen T (1992). Stimulatory and inhibitory effects of catecholamines
on DNA synthesis in primary rat hepatocyte cultures: role of alpha1- and
beta-adrenergic mechanisms. J Cell Physiol.

[27] El-Salhy M, Stenling R, Grimelius L (1993). Peptidergic innervation and endocrine cells in the
human liver. Scand J Gastroenterol.

[28] Stoyanova JL (2004). Relevance of mast cells and hepatic lobule
innervations to liver. Rom J Gastroenterol.

[29] Brauneis U, Gatmaitan Z, Arias IM: **Serotonin stimulates a Ca**^**2+**^**permeant nonspecific cation channel in hepatic endothelial cells.** *Biochem Biophys Res Commun* 1992, **186:**1560–1566. 10.1016/s0006-291x(05)81585-2 1380808

[30] Gatmaitan Z, Varticovski L, Ling L, Mikkelsen R, Steffan AM, Arias IM (1996). Studies on fenestral contraction in rat liver
endothelial cells in culture. Am J Pathol.

[31] Clavien PA (2008). Liver regeneration: a spotlight on the novel role of
platelets and serotonin. Swiss Med Wkly.

[32] Gooz M, Gooz P, Luttrell LM, Raymond JR (2006). 5-HT2A receptor induces ERK phosphorylation and
proliferation through ADAM-17 tumor necrosis factor-alpha-converting enzyme
(TACE) activation and heparin-bound epidermal growth factor-like growth factor
(HB-EGF) shedding in mesangial cells. J Biol Chem.

[33] Nebigil CG, Launay JM, Hickel P, Tournois C, Maroteaux L (2000). 5-hydroxytrypramine 2B receptor regulates cell-cycle
progression: cross-talk with tyrosine kinase pathways. Proc Natl Acad Sci U S A.

[34] Liu Y, Li M, Warburton RR, Hill NS, Fanburg BL (2007). The 5-HT transporter transactivates the PDGF beta
receptor in pulmonary artery smooth muscle cells. FASEB J.

[35] Mnie-Filali O, Faure C, Lambas-Senas L, El Mansari M, Belblidia H, Gondard E, Etievant A, Scarna H, Didier A, Berod A, Blier P, Haddjeri N (2011). Pharmacological blockade of 5-HT7 receptors as a
putative fast acting antidepressant strategy. Neuropsychopharmacology.

[36] Leon-Ponte M, Ahern GP, O’Connell PJ (2007). Serotonin provides an accessory signal to enhance
T-cell activation by signaling through the 5-HT7 receptor. Blood.

[37] Svejda B, Kidd M, Timberlake A, Harry K, Kazberouk A, Schimmack S, Lawrence B, Pfragner R, Modlin IM (2013). Serotonin and the 5-HT7 receptor: the link between
hepatocytes, IGF-1 and small intestinal neuroendocrine tumors. Cancer Sci.

[38] Mahe C, Loetscher E, Feuerbach D, Muller W, Seiler MP, Schoeffter P (2004). Differential inverse agonist efficacies of SB-258719,
SB-258741 and SB-269970 at human recombinant serotonin 5-HT7
receptors. Eur J Pharmacol.

[39] Lovell PJ, Bromidge SM, Dabbs S, Duckworth DM, Forbes IT, Jennings AJ, King FD, Middlemiss DN, Rahman SK, Saunders DV, Collin LL, Hagan JJ, Riley GJ, Thomas DR (2000). A novel, potent, and selective 5-HT7 antagonist:
(R)-3-(2-(2-(4-methylpiperidin-1-yl)ethyl)pyrrolidine-1-sulfonyl)phenol (SB
269970). J Med Chem.

[40] Foong JP, Bornstein JC (2009). 5-HT antagonists NAN-190 and SB 269970 block
αlpha2-adrenoceptors in the guinea pig. Neuroreport.

[41] Hagan JJ, Price GW, Jeffrey P, Deeks NJ, Stean T, Piper D, Smith MI, Upton N, Medhurst AD, Middlemiss DN, Riley GJ, Lovell PJ, Bromidge SM, Thomas DR (2000). Characterization of SB-269970-A, a selective 5-HT(7)
receptor antagonist. Br J Pharmacol.

[42] Hoffman BB, Dukes DF, Lefkowitz RJ (1981). Alpha-adrenergic receptors in liver membranes:
delineation with subtype selective radioligands. Life Sci.

[43] Bylund DB (1992). Subtypes of αlpha-1- and alpha-2- adrenergic
receptors. FASEB J.

[44] Seuwen K, Magnaldo I, Kobilka BK, Caron MG, Regan JW, Lefkowitz RJ, Pouyssegur J (1990). Alpha 2-adrenergic agonists stimulate DNA synthesis in
Chinese hamster lung fibroblasts transfected with a human alpha 2-adrenergic
receptor gene. Cell Regul.

[45] Chiesa IJ, Castillo LF, Luthy IA (2008). Contribution of alpha-2-adrenoceptors to the mitogenic
effect of catecholestrogen in human breast cancer MCF-7 cells. J Steroid Biochem Mol Biol.

[46] Bruzzone A, Pinero CP, Castillo LF, Sarappa MG, Rojas P, Lanari C, Luthy IA (2008). Alpha-2- adrenoceptor action on cell proliferation and
mammary tumour growth in mice. Br J Pharmacol.

[47] Shen SG, Zhang D, Hu HT, Li JH, Wang Z, Ma QY (2008). Effects of alpha-adrenoreceptor antagonists on
apoptosis and proliferation of pancreatic cancer cells in vitro. World J Gastroenterol.

[48] Kanno N, Lesage G, Phinizy JL, Glaser S, Francis H, Alpini G (2002). Stimulation of alpha2-adrenergic receptor inhibits
cholangiocarcinoma growth through modulation of Raf-1 and B-Raf
activities. Hepatology.

[49] Romero G, Pujol M, Pauwels PJ (2006). Reanalysis of constitutively active rat and human
5-HT7(a) receptors in HEK-293 F cells demonstates lack of silent properties for
reported neutral antagonists. Naunyn Schmiedebergs Arch Pharmacol.

[50] Forbes IT, Dabbs S, Duckworth DM, Jennings AJ, King FD, Lovell PJ, Brown AM, Collin L, Hagan JJ, Middlemiss DN, Riley GJ, Thomas DR, Upton N (1998). (R)-3,
N-dimethyl-N-[1-methyl-3-(4-methyl-piperidin-1-yl) propyl]benzenesulfonamide:
the first selective 5–HT7 receptor antagonist. J Med Chem.

[51] Perez-Garcia GS, Meneses A (2005). Effects of the potential 5-HT7 receptor agonist AS 19
in an autoshaping learning task. Behav Brain Res.

[52] Perez-Garcia G, Gonzalez-Espinosa C, Meneses A (2006). An mRNA expression analysis of stimulation and
blockade of 5-HT7 receptors during memory consolidation. Behav Brain Res.

[53] Sanin A, Brisander M, Rosqvist S, Mohell N, Malberg A, Johansson A (2004). 5-Aryl Substituted (*S*)-2-(Dimethylamino)-Tetralins Nover Serotonin 5HT 7 Receptor
Ligands. Proceedings of the 14th Camerino-Noord Symposium.

[54] Brenchat A, Rocasalbas M, Zamanillo D, Hamon M, Miguel Vela J, Romero L (2012). Assessment of 5-HT7 receptor agonists selectivity
using nociceptive and thermoregulation tests in knockout versus wild-type
mice. Adv Pharmacol Sci.

[55] Tijburg LB, Nyathi CB, Meijer GW, Geelen MJ (1991). Biosynthesis and secretion of triacylglycerol in rat
liver after partial hepatectomy. Biochem J.

[56] Rudnick DA (2005). Trimming the fat from liver
regeneration. Hepatology.

[57] Sumara G, Sumara O, Kim JK, Karsenty G (2012). Gut-derived serotonin is a multifunctional determinant
to fasting adaptation. Cell Metab.

[58] Nonogaki K (2000). New insights into sympathetic regulation of glucose
and fat metabolism. Diabetologia.

[59] Watts SW, Yang P, Banes AK, Baez M (2001). Activation of Erk mitogen-activated protein kinase
proteins by vascular serotonin receptors. J Cardiovasc Pharmacol.

[60] Lieb K, Biersack L, Waschbiisch A, Orlikowski S, Akundi ES, Candelario-Jalil E, Hull M, Fiebich BL (2005). Serotonin via 5-HT7 receptors activates p38
mitogen-activated protein kinase and protein kinase C epsilon resulting in
interleukin-6 synthesis in human U373 MG astrocytoma cells. J Neurochem.

[61] Norum JH, Hart K, Levy FO (2003). Ras-dependent ERK activation by the human G(s)-coupled
serotonin receptors 5-HT4(b) and 5-HT7(a). J Biol Chem.

[62] Klinger M, Kudlacek O, Seidel MG, Freissmuth M, Sexl V (2002). MAP kinase stimulation by cAMP does not require RAP1
but SRC family kinases. J Biol Chem.

[63] Enserink JM, Christensen AE, de Rooij J, van Triest M, Schwede F, Genieser HG, Doskeland SO, Blank JL, Bos JL (2002). A novel Epac-specific cAMP analogue demonstrates
independent regulation of Rap1 and ERK. Nat Cell Biol.

[64] Banes A, Florian JA, Watts SW (1999). Mechanisms of 5-Hydroxytryptamine (2A) receptor
activation of the mitogen-activated protein kinase pathway in vascular smooth
muscle. J Pharmacol Exp Ther.

